# 3D printing in medicine of congenital heart diseases

**DOI:** 10.1186/s41205-016-0004-x

**Published:** 2016-12-01

**Authors:** Shi-Joon Yoo, Omar Thabit, Eul Kyung Kim, Haruki Ide, Deane Yim, Anreea Dragulescu, Mike Seed, Lars Grosse-Wortmann, Glen van Arsdell

**Affiliations:** 1 Department of Diagnostic Imaging, University of Toronto, 555 University Avenue, Toronto, ON Canada; 2 Division of Cardiology - Department of Paediatrics, University of Toronto, 555 University Avenue, Toronto, ON Canada; 3 Division of Cardiovascular Surgery – Department of Surgery, Hospital for Sick Children, University of Toronto, 555 University Avenue, Toronto, ON M5G1X8 Canada; 4 3D HOPE (Human organ Printing and Engineering) Medical, 1008-65 Harbour Sqaure, Toronto, ON M5J2L4 Canada

**Keywords:** 3D printing, Congenital heart disease, Surgical simulation, Surgical training

## Abstract

Congenital heart diseases causing significant hemodynamic and functional consequences require surgical repair. Understanding of the precise surgical anatomy is often challenging and can be inadequate or wrong. Modern high resolution imaging techniques and 3D printing technology allow 3D printing of the replicas of the patient’s heart for precise understanding of the complex anatomy, hands-on simulation of surgical and interventional procedures, and morphology teaching of the medical professionals and patients. CT or MR images obtained with ECG-gating and breath-holding or respiration navigation are best suited for 3D printing. 3D echocardiograms are not ideal but can be used for printing limited areas of interest such as cardiac valves and ventricular septum. Although the print materials still require optimization for representation of cardiovascular tissues and valves, the surgeons find the models suitable for practicing closure of the septal defects, application of the baffles within the ventricles, reconstructing the aortic arch, and arterial switch procedure. Hands-on surgical training (HOST) on models may soon become a mandatory component of congenital heart disease surgery program. 3D printing will expand its utilization with further improvement of the use of echocardiographic data and image fusion algorithm across multiple imaging modalities and development of new printing materials. Bioprinting of implants such as stents, patches and artificial valves and tissue engineering of a part of or whole heart using the patient’s own cells will open the door to a new era of personalized medicine.

## Introduction

Congenital heart diseases are the most common significant birth defects with a live birth prevalence of 7.5 per 1000 [1]. Most congenital heart diseases causing significant hemodynamic and functional consequences require surgical repair. Modern imaging technologies including ultrasound, computed tomography (CT) and magnetic resonance (MR) provide accurate information regarding the anatomy and hemodynamic consequences of congenital heart disease. However, understanding of the surgical anatomy from the provided images requires a complicated process of mental reconstruction and can be often inadequate or wrong. In addition, communication among cardiologists, radiologists and surgeons is often difficult because of complex, diverse and controversial terms used in the description of congenital heart diseases, which may lead to misunderstanding of the surgical anatomy [2].

Virtual demonstration of the 3D structures on a 2D computer screen facilitates understanding of the complex anatomy. 3D printing takes a closer step toward the reality by providing the physical replicas out of the digital data processed for the virtual models. Medical applications of 3D printing have continuously been expanding [3, 4]. As far as we know, the 1^st^ paper on cardiac application of 3D printing was published in 2000 by Binder et al [5]. In the last 15 years, 3D printing has increasingly been used in the diagnosis, management and education of congenital heart diseases [5–34]. This review paper will introduce the applicable imaging techniques, post-processing and printing procedures, current applications and limitations, and future directions of 3D printing in the medicine of congenital heart diseases.

## Review

### Applicable medical imaging techniques

Any medical images acquired in 3D demonstrating the blood pool distinct from the myocardium and vessel wall can be used for 3D printing (Table 1). Ideally, electrocardiographic (ECG) gating and breath-holding or respiration navigation is required to avoid artifact from cardiac and respiratory motion. However, the images obtained without ECG-gating and/or free breathing are still applicable for 3D printing if it is not aimed to show small structures such as coronary arteries. The volume data with isotropic resolution is preferred.

**Table 1:** Imaging techniques applicable for 3D printing of heart models

Imaging Modality	Imaging Techniques
Computed tomography (CT)	● ECG-gated breath-held contrast-enhanced angiography ● Non-ECG-gated contrast-enhanced angiography
Magnetic resonance (MR)	● Non-contrast 3D SSFP (steady state free precession) imaging ● Non-ECG-gated 3D FLASH (fast low angle shot) angiography using gadolinium-based extracellular contrast agent ● ECG-gated respiration-navigated 3D IR (inversion recovery) FLASH angiography using gadolinium-based blood pool contrast agent (Gadofoveset: ABLAVAR®, Lantheus Medical Imaging, Inc. MA, USA) ● ECG-gated respiration-navigated 4D MUSIC (multiphase steady-state imaging with contrast enhancement) using ultra-small supermagnetic iron oxide (USPIO: Ferumoxyol, AMAG Pharmaceuticals, Lexington, MA, USA)
Ultrasound	● 3D grey-scale echocardiography ● 3D color or power Doppler echocardiography
X-ray angiography	● Rotational CT angiography

ECG-gated CT angiograms provide a spatial resolution of 0.3–0.7 mm and are the most commonly used images among currently applicable imaging modalities in 3D printing of cardiovascular structures. In CT angiography, it is important to time the scanning when all cardiac chambers are homogeneously enhanced. It is also important to inject a generous amount of saline chaser to minimize the artifact from undiluted contrast medium remaining in the superior or inferior vena cava and its tributaries. Although MR angiograms may provide <1 mm spatial resolution, high resolution imaging is at the expense of significant compromise in signal-to-noise ratio. If there is no significant stenotic lesion or valvular regurgitation that cause artifact from turbulent flow, non-contrast 3D SSFP (steady state free precession) imaging provides the images of sufficient quality. However, contrast-enhanced angiography is required in most cases with congenital heart disease. In conventional MR angiography using an extracellular contrast agent, ECG-gating is hardly applicable and a degree of artifact from cardiac motion is unavoidable. ECG-gated and respiration-navigated 3D FLASH (fast low angle shot) angiography using blood-pool contrast agent (Gadofoveset: ABLAVAR®, Lantheus Medical Imaging, Inc. MA, USA) provides excellent images with homogeneous distribution of contrast medium and no significant artifact from turbulent flow [35]. Most recently, ultrasmall superparamagnetic iron oxide (USPIO: Ferumoxyol, AMAG Pharmaceuticals, Lexington, MA, USA) that is used for treatment of iron deficiency anemia has been tried for angiography in children with excellent results [36], and can certainly be applied for 3D printing.

Ultrasound is not an ideal imaging modality for 3D printing because of the limited access windows for imaging and abundant artifacts from bones and air. However, certain parts of the heart such as atrial and ventricular septa can be imaged appropriately for 3D printing [3, 4, 25, 26, 37]. Although the results are not satisfactory, cardiac valve leaflets can also be imaged and printed with ultrasound data. Lastly, rotational CT angiograms obtained from modern x-ray angiographic equipment can be used for 3D printing [38].

### Postprocessing of image data

**Fig. 1:**
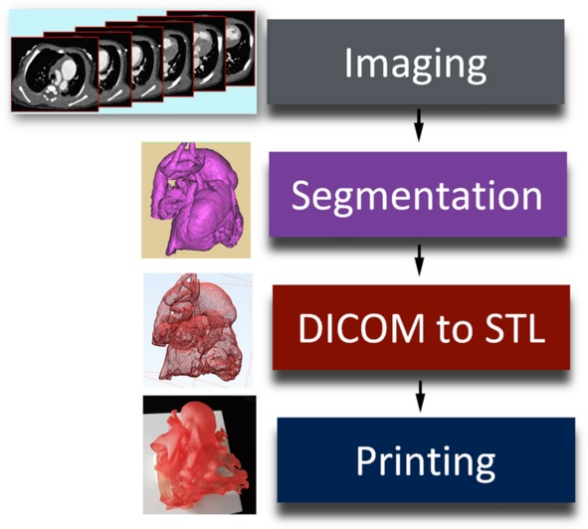
Diagram showing the steps in data processing for 3D printing. DICOM, digital imaging and communication in medicine; STL, stereolithography, standard tessellation language or standard triangle language

The postprocessing procedure includes: 1) segmentation, 2) conversion of the DICOM (**D**igital **I**maging and **Co**mmunication in **M**edicine) file to the STL (**St**ereo**l**ithography or **S**tandard **T**essellation **L**anguage) or other file fomat for 3D printing, and 3) computer aided design (CAD) (Fig. 1).

**Fig. 2:**
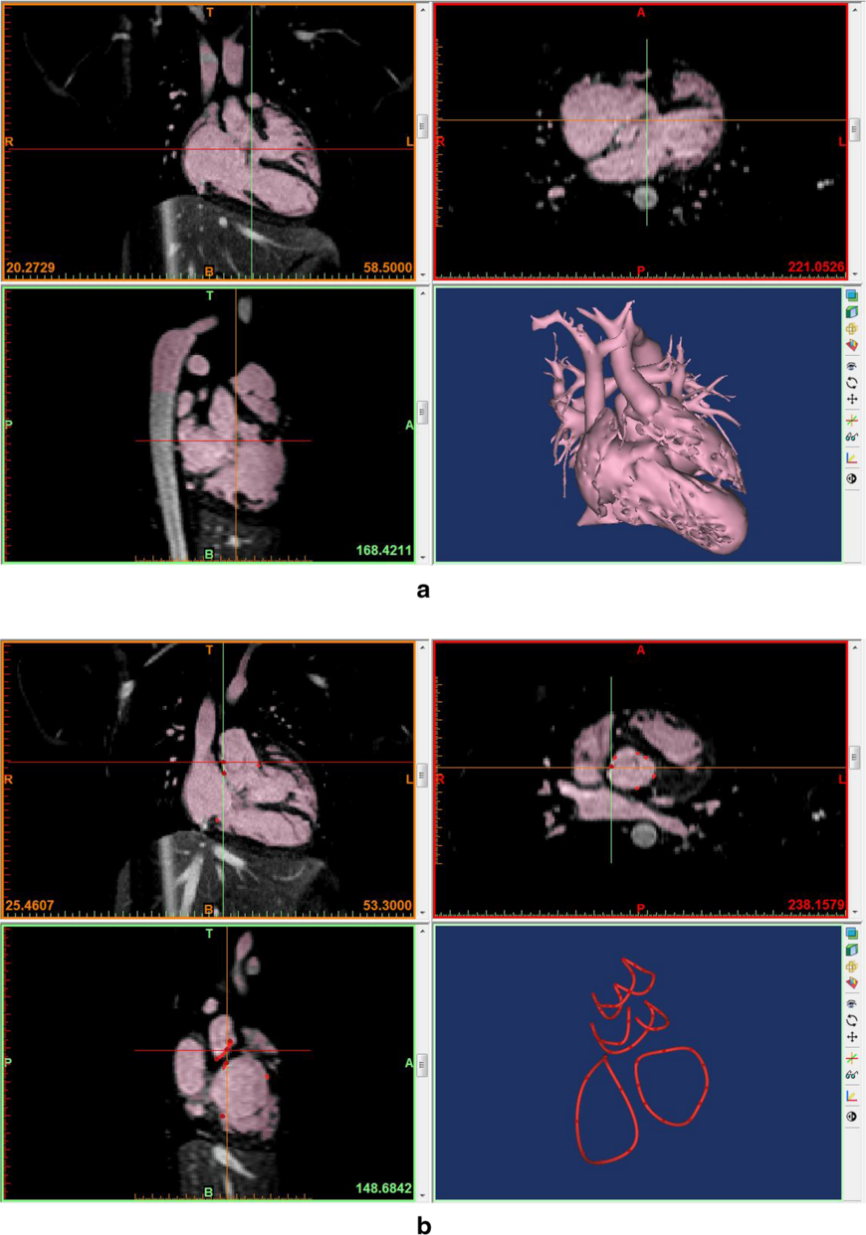
Segmentation process in a commercially available software program (Mimics®, Materialise, Leuven, Belgium) using magnetic resonance angiograms from a patient with congenitally corrected transposition of the great arteries with a ventricular septal defect. **a** Segmentation using thresholding and manual edition with a volume rendered image on the right lower panel. **b** Linear representation of the cardiac valvar attachments. A few points of attachment sites of each cardiac valve were marked and connected using a tool called “spline”

**Fig. 3:**
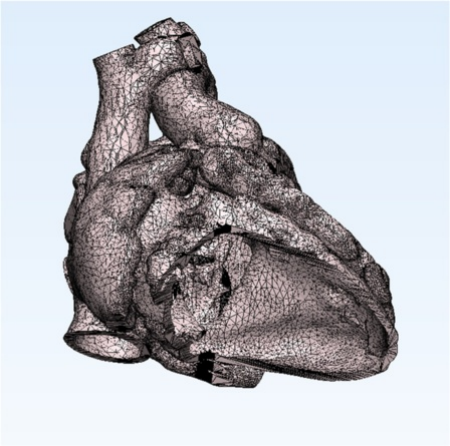
Surface geometry in STL (**St**ereo**l**ithography or **S**tandard **T**essellation **L**anguage) file from the patient shown in Fig. 2. The surface of the object is divided into a number of triangles without any gap and overlap

The blood pool is segmented using thresholding algorithm with manual adjustment (Fig. 2). The better the image quality, the easier the segmentation process. When the boundary between the blood pool and the myocardium or vessel wall is not readily recognizable by automated thresholding, extensive manual work using drawing, erasing and regional thresholding tools and interpolation of the data between the slices are required. The manual work requires in depth understating of normal and pathological anatomy as well as its appearance on cross-sectional imaging. Once segmentation is completed, the 3D volume data is converted to a file format for 3D printing (Fig. 3). A few file formats including STL, VRML (**V**irtual **R**eality **M**odeling **L**anguage), AMF (**A**dditive **M**anufacturing **F**ile Format) and OBJ (**Obj**ect) file are available for 3D printing. STL is the most commonly used file format providing a single color for 3D rendering (https://doi.org/en.wikipedia.org/wiki/STL_(file_format). OBJ is a simple data format that represents 3D geometry alone (https://doi.org/en.wikipedia.org/wiki/Object_file). VRML and AMF provide multiple color and material options (https://doi.org/en.wikipedia.org/wiki/VRML, https://doi.org/en.wikipedia.org/wiki/Additive_Manufacturing_File_Format). The file format conversion process includes a user-defined number of iterations for smoothing of the surface of the object. The lower number of iteration provides the models that are close to what are in the original image data, while the surface of the model may appear rough. The higher number of iteration provides a smoother surface of the model, while the detail of the surface anatomy is compromised to a certain extent. The operator needs to define the optimum number of iteration and smoothing factor according to the purposes of 3D printing. As the current imaging technologies do not provide good images of the cardiac valves, it is advisable to demarcate the annuli of the cardiac valves on the model. By marking a few points on the attachment of the valves to the wall using CAD program, an interpolated line of valve insertion is created and assigned a thickness (Figs. 2b and 3). The graphically designed annuli of the cardiac valves are then added to the model.

**Fig. 4:**
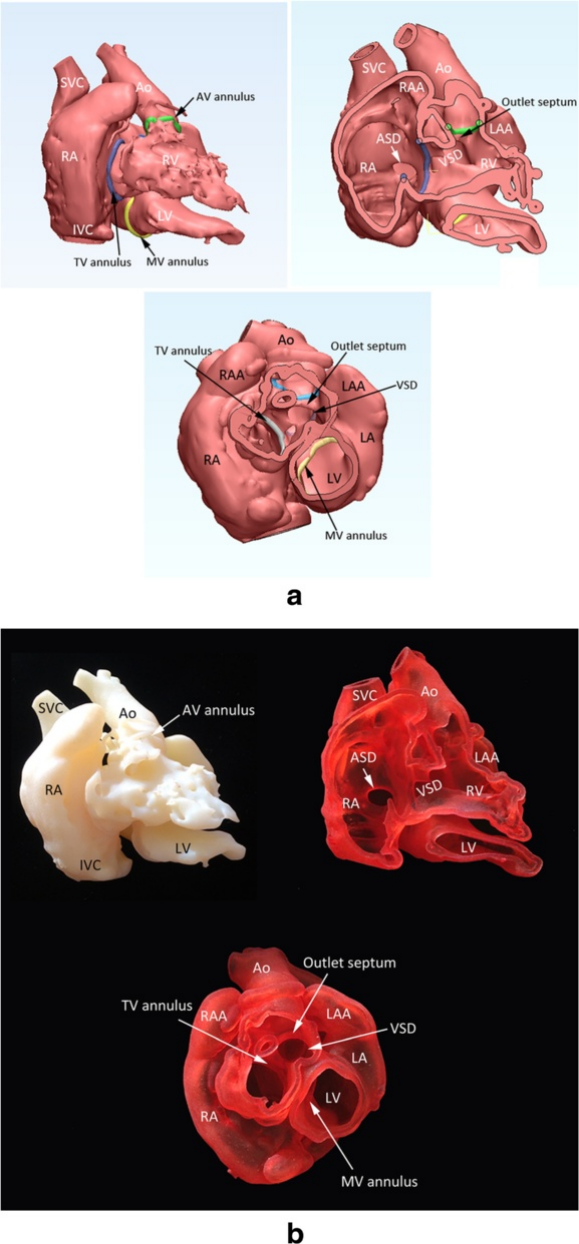
Screen-display of the STL files (**a**) and photographs of the corresponding models (**b**) in a case with so-called twisted or criss-cross heart with transposition of the great arteries and a ventricular septal defect (VSD). Cast model (top left), wall model after removal of the anterior free wall of the right atrium and right and left ventricles (top right), and wall model with the apical two thirds of the ventricles removed (bottom) are shown. The cardiac valve annuli are marked by the spline curves that were graphically designed as shown in Fig. 2B. Ao, aorta; ASD, atrial septal defect; AV, aortic valve; IVC, inferior vena cava; LA, left atrium; LAA, left atrial appendage; LV, left ventricle; MV, mitral valve; SVC, superior vena cava; TV, tricuspid valve

**Fig. 5:**
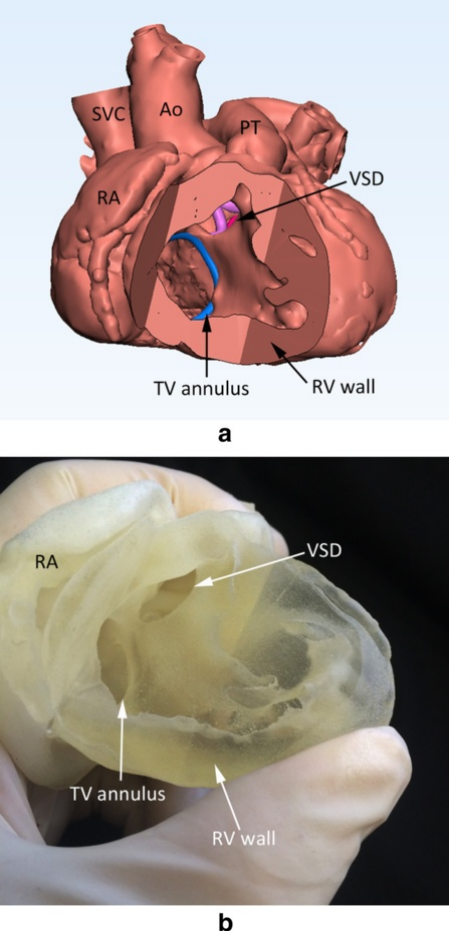
Screen-display (**a**) and photograph (**b**) of the 3D print model of the heart with tetralogy of Fallot. The full thickness of the myocardium was carefully segmented with both endocardial and epicardial boundaries delineated by thresholding and manual editing. Although it is considered ideal, the postprocessing was time consuming and the model is stiff to be used for surgical simulation. Ao, aorta; PT, pulmonary trunk; SVC, superior vena cava; TV, tricuspid valve; RA, right atrium; RV, right ventricle; VSD, ventricular septal defect

**Fig. 6:**
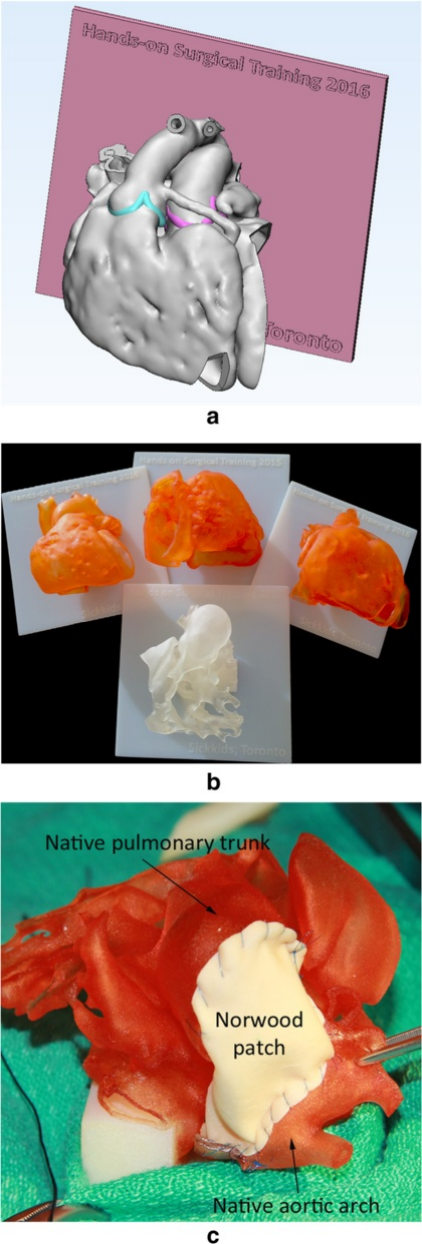
Models for surgical practice or training. **a** Screen-display of the STL file shows the heart mounted on a graphically designed platform. **b** Four example models for surgical training. **c** A 3D print model with hypoplastic left heart syndrome on which a surgeon underwent a Norwood procedure

For the assessment of congenital heart diseases, two types of heart models are valuable: cast models of blood pool and wall models for endocardial surface representation (Fig. 4). Cast models provide an excellent overview of the anatomy. Wall models provide detailed information regarding the endocardial surface anatomy. It is ideal to have the entire wall of the heart and vessels represented in the 3D print models (Fig. 5). To achieve this goal, both inner and outer boundaries of the cardiac cavities and vessels (endocardial and epicardial surfaces for the heart) should be delineated. Although achievable, it is a time consuming process to delineate the outer boundary of the wall as the signal intensities of the myocardium and vessels are not distinctively different from those of the adjacent mediastinal tissues. Furthermore, a complete wall model requires a large amount of expensive print material, while it is stiff and heavy. As the surgeons operate mostly on the inside of the heart, it is generally sufficient if the endocardial surface anatomy is accurately shown on the models. The endocardial surface anatomy can be represented by graphically adding a shell on the surface of the cavity cast that is clearly and distinctly definable. The result is a negative of the cast model with a wall of an arbitrary thickness (Fig. 4a). In order to visualize the inner surfaces of the heart and vessels, a few windows are made on the shell. For surgical practice, the entry window for surgical procedure is opened and the model is mounted on the plate to provide a stable position and to restrict movement of the model during the procedure (Fig. 6). However, such shelling process is not applicable for representation of the lumens of small and tightly packed vessels such as peripheral pulmonary arteries and veins.

### 3D printing process

The STL files are loaded to the software program of the 3D printer and the materials are assigned to the files for printing. Most commercially available 3D printers are designed for building rigid plastic models, while a few printers are capable of building models with soft rubber-like materials. Ideally, the printing material should have the physical properties such as consistency, elasticity, tensile strength, tear resistance and memory capacity that are similar to those of human soft tissue. Among the existing 3D printers, the printers using polyjet technology and photopolymer resin materials (Objet Connex Series printer and TangoPlus FullCure resin, Stratasys Ltd, Minnesota, USA, and Projet 5500X printer and Visijet CE NT-Elastomeric Natural resin, 3DSystems, Rockhill, USA) provide the physical properties of the printed models closest to those of human soft tissue, allowing simulated surgical and interventional procedures. If the purpose of 3D printing is for demonstration of the anatomy of the heart, any commercially available printer with an acceptable print resolution can be used.

With current 3D printing technology, 3D printing typically takes 3–10 hours to build a single piece of the heart model depending on its size. After the model is completely built, it is harvested and the supporting material and/or the unused print materials are washed out with a waterjet, blown away with an airjet or melted down with chemicals and water. Depending on the materials used and the complexity of the geometry, this cleaning process takes a few minutes to an hour. Powder-based models require curing with chemicals and heat. Although some printers build the models with multiple colors, others provide limited color options. When the printed model is white or faintly colored, one may want to dye the model with a slightly dark color for improved perceptual representation of the complex surface anatomy.

### Current applications

#### Planning and simulation for surgical and interventional procedures [9, 13–21, 23, 27–31]

Preoperative assessment with 3D print models reduces the degree of uncertainty as regards to the patient’s specific anatomy. In selected cases, 3D printing can contribute to an improved outcome as precise preoperative understanding of the complex anatomy may obviate or shorten lengthy exploration, and therefore operation and cardiopulmonary bypass time can be reduced [31]. Surgical procedure on a patient with congenital heart disease is usually performed through a midline sternotomy or lateral thoracotomy and a small incision in the wall of an atrium, an arterial trunk or, rarely, a ventricle. As the patient’s thorax and heart sizes are small in children, the actual surgical scene is difficult to inspect especially from the assistant’s position during the surgery. If the sterilized models showing the important surgical anatomy of the patient’s heart were given to the surgical team, the primary operator’s procedure would be facilitated with precise and streamlined assistance from the assistants [18]. In addition, 3D print models made of flexible material can be used for practice surgery before the real operation.

**Fig. 7:**
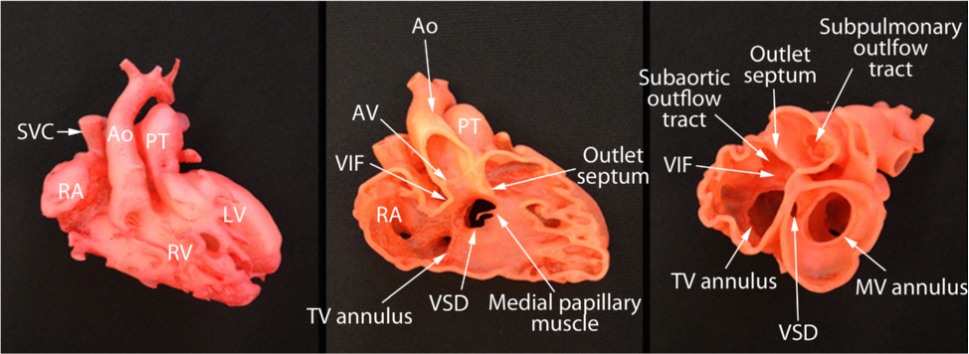
Photographs of the 3D print models of a case with double outlet right ventricle. Although echocardiograms showed that the ventricular septal defect (VSD) is remote from both arterial valves, 3D models show that the VSD is able to be routed to the aortic valve allowing biventricular repair. Ao, aorta; AV, aortic valve; LA, left atrium; LV, left ventricle; MV, mitral valve; SVC, superior vena cava; TV, tricuspid valve; VIF, ventriculoinfundibular fold

For preoperative assessment of congenital heart diseases, we typically make three models for each case: a cast model of blood pool for the overview, a wall model with the atrial and ventricular free wall partly removed for the atrial and ventricular septal anatomy, and a wall model with the apical halves to two thirds of the ventricles removed for the anatomy of the bases of the ventricles (Fig. 4). As discussed, a model mounted on a plate can be provided for preoperative surgical practice (Fig. 6). Table 2 lists the indications for 3D printing service in patients with congenital heart disease at our institution since 2009. The most common indication was double outlet right ventricle where the feasibility of intraventricular baffling procedure should be accurately assessed before undergoing biventricular repair (Fig. 7). In this regard, 3D print models certainly provide the surgeons with clear and undisputable information. Although it is rare, so-called twisted or criss-cross heart is an important indication for 3D printing (Fig. 4). In these cases, the spatial relationship of the cardiac chambers and great arteries and overall intracardiac anatomy are difficult to understand and explain. With the 3D print models in the observer’s hands, complex anatomy can be understood instantaneously and requires no explanation using ambiguous terminology introduced in the description of this particular pathology [39].

**Table 2:** Indications for 3D printing since 2009 (70 cases)

Congenital heart disease	Cases number
Double outlet right or left ventricle	37
Transposition of the great arteries, complete and congenitally corrected	8
Criss-cross heart or superoinferior ventricles	4 (3*)
Heterotaxy with complex heart disease	9 (7*)
Complex form of ventricular septal defect	6
Anomalous pulmonary venous connection	3
Complex tetralogy of Fallot	2
Coarctation of the aorta	1

Cases marked with asterisk also had double outlet right ventricle

**Fig. 8:**
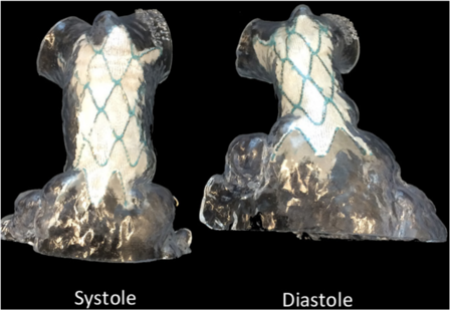
Photographs of the 3D print models of right ventricular outflow tract obtained in systole and diastole from a patient with severe pulmonary regurgitation after repair of tetralogy of Fallot. The fitness of the stent in the outflow tract was tested for both systole and diastole before the procedure

For the surgical simulation, the print materials are still far from ideal and the representation of the cardiac valves is limited. The print material does not represent tissue properties of myocardium and enocardial and epicardial linings, limiting assessment of the tissue response to the surgical procedure or deployment of medical devices. In our limited experience, however, the surgeons find the models suitable for practicing surgical simulation procedures such as closure of the septal defects, application of the baffles within the ventricles, reconstructing the aortic arch, and arterial switch procedure (Fig. 6). 3D print models are also used for interventional procedures to test whether the size and shape of the device would fit the patient’s specific anatomy and to practice the intended procedure [13, 14] (Fig. 8).

#### Contemporary morphology teaching with 3D print models [32–34]

Traditionally, pathological specimens removed from the patients during autopsy or heart transplantation are used for cardiac morphology teaching. Although the specimens are valuable educational resources, they are scarce and do not represent the whole spectrum of pathology. With improvement of the surgical and medical management of congenital heart diseases and changing concept on human right issues regarding retention of the removed human organs in the pathology laboratory, fewer specimens will be available. On the other hand, the existing specimens are exposed to wear and tear. 3D print models are great educational resources (Figs. 4 and 7) [33, 34]. By using the living patients’ imaging data, almost entire varieties of congenital heart diseases can be covered with 3D print models. The pathological features can be demonstrated in any desired planes or views. Any number of models can be reproduced and shared, and access to the models is not limited. With current technology, however, the valve tissues and myocardial pathology are hardly reproducible. Despite such a limitation, contemporary morphology teaching sessions using 3D printed educational model sets have increasingly been introduced in international and national meetings in the last few years.

3D print models are helpful in education of the patients and their parents [32]. The patient’s cardiovascular pathology and the intended or previously performed surgical or interventional procedures are easy to understand when they are explained using 3D print models.

#### Hands-on surgical training (HOST) [12, 13, 22, 23, 25]

Learning surgical techniques in congenital heart disease is challenging. As discussed, the size of the heart in children is usually small and the access routes for the procedure are limited for observation. In addition, the rarity of certain congenital heart diseases further limits the opportunity to learn and to improve the surgical skills. 3D printed models are great resources for surgical training (Fig. 6). The supervisors can take unlimited time in showing their procedures. The trainees can take enough time in learning and practicing surgical procedures and repeat the procedures until they feel confident. To the experienced surgeons, 3D print models can be used for development of the new procedures or to improve their surgical skills for rare diseases. In a personal communication, one of our senior surgeons commented that it usually takes a few years for the surgeons to learn how to do the Norwood operation in hypoplastic left heart syndrome and that he should have been able to learn the procedure overnight if 3D print models of a few cases with different pathologic variations would have been available for practice. We organized or supported three HOST courses in the last 12 months. All three courses were successful with high audience satisfaction [Yoo SJ, Spray T, Austine E, van Arsdell GS, et al. Hands-on surgical training (HOST) on congenital heart surgery using 3D print models. In preparation]. We strongly advise the surgeons and trainees to practice their surgical skills on 3D print models first before performing the specific operation on the patients.

### Current limitations

The limitations of 3D printing occur in all stages, during imaging, postprocessing and printing. Precise representation of any moving anatomical structures requires high spatial and temporal resolutions. With currently available imaging technologies, it is difficult to image the fine moving structures such as valve leaflets and chordae tendinae with the image quality sufficient enough for 3D printing. These fine structures are important as the abnormalities in these structures are usually associated with significant hemodynamic functional consequences and require delicate surgical repair. As discussed, 3D printing has significant limitations in using the data from ultrasound which is the primary and cheapest imaging technology in cardiac imaging.

Segmentation of the required structures in postprocessing software programs primarily relies on thresholding of signal intensities. When the adjacent structures do not have distinctly different signal intensities allowing automatic boundary detection, extensive time-consuming manual editing is required and the accuracy is significantly compromised.

It is ideal to print the heart using flexible materials with the physical properties similar to that of the human myocardium and valve tissues, especially when practice procedures were to be performed on the models. There are only a few flexible print materials, each of them being able to be used on a specific type of printer only. The surgeons find the models made of flexible material are more difficult to be sewn and easily torn or cut through as compared to real human myocardium or vessels.

The most important limiting factor in applications of 3D printing in patient care and teaching medical professionals is its high cost rather than its utility. The available software programs, printers and printing materials are expensive. Postprocessing is labor-intensive and often requires the hands of experienced imagers. Although 3D printing is also called rapid prototyping, it is fundamentally a time consuming process to build any object by adding numerous layers. The expenses per case can be unbearably high for small programs.

Although most 3D printers are provided with the company’s own specification regarding print resolution, reports on its reproducibility and accuracy in human applications are scarce [9, 19, 31] and prospective studies in larger cohorts are required. Nonetheless, it is of no doubt that 3D models are very helpful in understanding the complex morphology and spatial relationship among the structures. However, the models should be carefully reviewed in conjunction with the standard imaging findings as certain degrees of distortion and abbreviation of the information are inevitable during postprocessing and printing.

As have been the cases for any other procedures in their early developing phases when the objective data are lacking or scarce, it will take time for the insurance and its governing organizations to recognize 3D printing as a standard medical procedure and provide reimbursement for the service. In this respect, prospective clinical trials on use of 3D printing in congenital heart disease surgery and surgical training are crucially important to prove the cost-benefit of this newly developing technology.

### Future direction

Of little doubt, 3D printing will be widely used in the medicine of congenital heart diseases. Hands-on surgical training will gradually become an essential component in the surgical training programs. For rare and complex surgical procedures, hands-on training on the 3D print models will be the prerequisite requirement for performing the procedure on the patients.

**Fig. 9:**
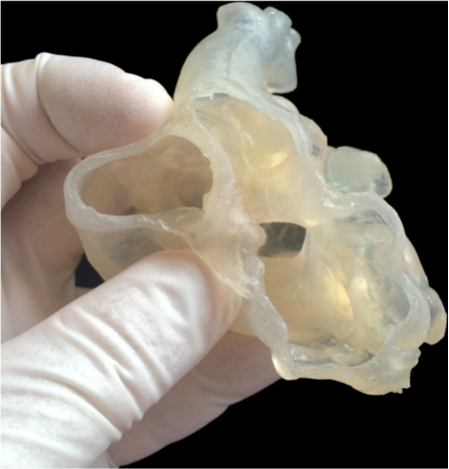
Photograph of a model made of silicone using injection molding technique

Currently available print materials are not satisfactory for surgical practice. High quality silicone appears closest to the myocardial tissue and best suited for surgery. Currently, silicon models can be made using injection molding technology where silicone is infused into the 3D printed mold (Fig. 9). Shiraishi et al used urethane to produce models with rubber-like consistency using molding technique [20]. Although 3D printers using silicon as the printing material are not available, a few commercial companies have recently announced the plans for release of silicone-based printer in the near future (Wacker Cheme AG, Munchen, Germany and Picisma Ltd., Sheffield, UK) (https://doi.org/3dprint.com/88316/wacker-3d-printed-silicone/, https://doi.org/www.picsima.com/#!3d-printing-silicone/cjg9).

As discussed, each imaging modality has its own strengths and weaknesses. Both contrast-enhanced CT and MR are excellent in visualization of the blood pool, while ultrasound is far superior to CT and MR in the demonstration of the anatomy and function of the cardiac valves and chordae tendinae. Image fusion is to improve the image content by combining useful information from multiple imaging modalities [40]. The process requires precise image registration process for spatial coordination between the images from different imaging modalities and mathematical algorithm for combining data from different sources. Image fusion technology will certainly enhance the image quality by compensating the weaknesses of each imaging modality and reduce the extent of artifact.

3D printing can be used for personalized implants such as stents, surgical patches and artificial valves [41, 42]. 3D printing has also been experimentally used for tissue engineering such as cardiac valves. Using 3D printing, a scaffold is printed and the cardiac progenitor cells are laid down with matrix for growth [43–45]. While a clinically viable product has not yet been fabricated, tissue printing eventually will allow fabrication of the implants made of the patient’s own stem cells and possibly obviate drug testing on animals by testing on bioprinted human tissues or organs [26].

## Conclusions

3D printing has found its niche applications in the medicine of congenital heart diseases. 3D print models allow instantaneous understanding of any complex anatomy and simulation or hands-on training of surgical and interventional procedures. It has made a small revolution in teaching and surgical practice. We expect that Hands-on surgical training (HOST) will soon become a mandatory component of the congenital heart disease surgery programs. 3D printing will expand its utilization with further improvement of imaging and printing technologies and development of new printing materials. Bioprinting and tissue engineering will open the door to the new era of personalized medicine.

## Abbreviations

CAD: computer aided design
CT: computed tomography
DICOM: digital Imaging and communication in medicine
ECG: electrocardiography
HOST: hands-on surgical training
MR: magnetic resonance
SSFP: steady state free precession
STL: stereolithography or standard tessellation language

## References

[1] Dolk H, Loane M, Garne E (2011). Congenital heart defects in Europe: prevalence and perinatal mortality, 2000 to 2005. Circulation.

[2] Giroud JM, Jacobs JP, Spicer D, Backer C, Martin GR, Franklin RC, Béland MJ, Krogmann ON, Aiello VD, Colan SD, Everett AD, William Gaynor J, Kurosawa H, Maruszewski B, Stellin G, Tchervenkov CI, Walters HL, Weinberg P, Anderson RH, Elliott MJ (2010). Report from the international society for nomenclature of paediatric and congenital heart disease: creation of a visual encyclopedia illustrating the terms and definitions of the international pediatric and congenital cardiac code. World J Pediatr Congenit Heart Surg.

[3] Mitsouras D, Liacouras P, Imanzadeh A, Giannopoulos AA, Cai T, Kumamaru KK, George E, Wake N, Caterson EJ, Pomahac B, Ho VB, Grant GT, Rybicki FJ (2015). Medical 3D printing for the radiologist. Radiographics.

[4] Matsumoto JS, Morris JM, Foley TA, Williamson EE, Leng S, McGee KP, Kuhlmann JL, Nesberg LE, Vrtiska TJ (2015). Three-dimensional physical modeling: applications and experience at mayo clinic. Radiographics.

[5] Binder TM, Moertl D, Mundigler G, Rehak G, Franke M, Delle-Karth G, Mohl W, Baumgartner H, Maurer G (2000). Stereolithographic biomodeling to create tangible hard copies of cardiac structures from echocardiographic data: in vitro and in vivo validation. J Am Coll Cardiol.

[6] Gilon D, Cape EG, Handschumacher MD, Song JK, Solheim J, VanAuker M, King ME, Levine RA (2002). Effect of three-dimensional valve shape on the hemodynamics of aortic stenosis: three-dimensional echocardiographic stereolithography and patient studies. J Am Coll Cardiol.

[7] Mottl-Link S, Boettger T, Krueger JJ, Rietdorf U, Schnackenburg B, Ewert P, Berger F, Nagel E, Meinzer HP, Juraszek A, Kuehne T, Wolf I (2005). Images in cardiovascular medicine. Cast of complex congenital heartmalformation in a living patient. Circulation.

[8] Noecker AM, Chen JF, Zhou Q, White RD, Kopcak MW, Arruda MJ, Duncan BW (2006). Development of patient-specific three-dimensional pediatric cardiac models. ASAIO J.

[9] Ngan EM, Rebeyka IM, Ross DB, Hirji M, Wolfaardt JF, Seelaus R, Grosvenor A, Noga ML (2006). The rapid prototyping of anatomic models in pulmonary atresia. J Thorac Cardiovasc Surg.

[10] Greil GF, Wolf I, Kuettner A, Fenchel M, Miller S, Martirosian P, Schick F, Oppitz M, Meinzer HP, Sieverding L (2007). Stereolithographic reproduction of complex cardiac morphology based on high spatial resolution imaging. Clin Res Cardiol.

[11] Greil GF, Kuettner A, Flohr T, Grasruck M, Sieverding L, Meinzer HP, Wolf I (2007). High-resolution reconstruction of a waxed heart specimen with flat panel volume computed tomography and rapid prototyping. J Comput Assist Tomogr.

[12] Kim MS, Hansgen AR, Wink O, Quaife RA, Carroll JD (2008). Rapid prototyping: a new tool in understanding and treating structural heart disease. Circulation.

[13] Armillotta A, Bonhoeffer P, Dubini G, Ferragina S, Migliavacca F, Sala G, Schievano S (2007). Use of rapid prototyping models in the planning of percutaneous pulmonary valved stent implantation. Proc Inst Mech Eng H.

[14] Schievano S, Migliavacca F, Coats L, Khambadkone S, Carminati M, Wilson N, Deanfield JE, Bonhoeffer P, Taylor AM (2007). Percutaneous pulmonary valve implantation based on rapid prototyping of right ventricular outflow tract and pulmonary trunk from MR data. Radiology.

[15] Kim MS, Hansgen AR, Carroll JD (2008). Use of rapid prototypingin the care of patients with structural heart disease. Trends Cardiovasc Med.

[16] Jacobs S, Grunert R, Mohr FW, Falk V (2008). 3D-Imaging of cardiac structures using 3D heart models for planning in heart surgery: a preliminary study. Interact Cardiovasc Thorac Surg.

[17] Mottl-Link S, Hübler M, Kühne T, Rietdorf U, Krueger JJ, Schnackenburg B, De Simone R, Berger F, Juraszek A, Meinzer HP, Karck M, Hetzer R, Wolf I (2008). Physical models aiding in complex congenital heart surgery. Ann Thorac Surg.

[18] Sodian R, Weber S, Markert M, Loeff M, Lueth T, Weis FC, Daebritz S, Malec E, Schmitz C, Reichart B (2008). Pediatric cardiac transplantation: three dimensional printing of anatomic models for surgical planning of heart transplantation in patients with univentricular heart. J Thorac Cardiovasc Surg.

[19] Riesenkampff E, Rietdorf U, Wolf I, Schnackenburg B, Ewert P, Huebler M, Alexi-Meskishvili V, Anderson RH, Engel N, Meinzer HP, Hetzer R, Berger F, Kuehne T (2009). The practical clinical value of three-dimensional models of complex congenitally malformed hearts. J Thorac Cardiovasc Surg.

[20] Shiraishi I, Yamagishi M, Hamaoka K, Fukuzawa M, Yagihara T (2010). Simulative operation on congenital heart disease using rubber-like urethane stereolithographic biomodels based on 3D datasets of multislice computed tomography. Eur J Cardiothorac Surg.

[21] Farooqi KM, Nielsen JC, Uppu SC, Srivastava S, Parness IA, Sanz J, Nguyen K (2015). Use of 3-dimensional printing to demonstrate complex intracardiac relationships in double-outlet right ventricle for surgical planning. Circ Cardiovasc Imaging.

[22] Costello JP, Olivieri LJ, Krieger A, Thabit O, Marshall MB, Yoo SJ, Kim PC, Jonas RA, Nath DS (2014). Utilizing three-dimensional printing technology to assess the feasibility of high fidelity synthetic ventricular septal defect models for simulation in medical education. World J Pediatr Congenit Heart Surg.

[23] Olivieri L, Krieger A, Chen MY, Kim P, Kanter JP (2014). 3D heart model guides complex stent angioplasty of pulmonary venous baffle obstruction in a Mustard repair of D-TGA. Int J Cardiol.

[24] Yoo SJ, Lo Rito M, Seed M, Grosse-Wortmann L (2014). Magnetic resonance imaging as a decision-making tool in congenital heart disease surgery. Operative Tech Thorac Cardiovascul Surg.

[25] Olivieri LJ, Krieger A, Loke YH, Nath DS, Kim PC, Sable CA (2015). Three-dimensional printing of intracardiac defects from three-dimensional echocardiographic images: feasibility and relative accuracy. J Am Soc Echocardiogr.

[26] Farooqi KM, Sengupta PP (2015). Echocardiography and three-dimensional printing: sound ideas to touch a heart. J Am Soc Echocardiogra.

[27] Costello JP, Olivieri LJ, Su L, Krieger A, Alfares F, Thabit O, Marshall MB, Yoo SJ, Kim PC, Jonas RA, Nath DS (2015). Incorporating three-dimensional printing into a simulation-based congenital heart disease and critical care training curriculum for resident physicians. Congenit Heart Dis.

[28] Kiraly Laszlo, Tofeig Magdi, Jha Neerod Kumar, Talo Haitham (2015). Three-dimensional printed prototypes refine the anatomy of post-modified Norwood-1 complex aortic arch obstruction and allow presurgical simulation of the repair. Interactive CardioVascular and Thoracic Surgery.

[29] Valverde I, Gomez G, Coserria JF, Suarez-Mejias C, Uribe S, Sotelo J, Velasco MN, Santos De Soto J, Hosseinpour AR, Gomez-Cia T (2015). 3D printed models for planning endovascular stenting in transverse aortic arch hypoplasia. Catheter Cardiovasc Interv.

[30] Valverde I, Gomez G, Gonzalez A, Suarez-Mejias C, Adsuar A, Coserria JF, Uribe S, Gomez-Cia T, Hosseinpour AR (2015). Three-dimensional patient-specific cardiac model for surgical planning in Nikaidoh procedure. Cardiol Young.

[31] Ma XJ, Tao L, Chen X, Li W, Peng ZY, Chen Y, Jin J, Zhang XL, Xiong QF, Zhong ZL, Chen XF (2015). Clinical application of three-dimensional reconstruction and rapid prototyping technology of multislice spiral computed tomography angiography for the repair of ventricular septal defect of tetralogy of Fallot. Genet Mol Res.

[32] Biglino G, Capelli C, Wray J, Schievano S, Leaver LK, Khambadkone S, Giardini A, Derrick G, Jones A, Taylor AM (2015). 3D-manufactured patient-specific models of congenital heart defects for communication in clinical practice: feasibility and acceptability. BMJ Open.

[33] Yoo SJ, Thabit O, Lee W, Goo HW, van Arsdell GS (2013) Double outlet right ventricle in your hands. Web publication. https://doi.org/imib-chd.com/wp-content/uploads/morphology/1-dorv-in-your-hands/DORV%20IN%20YOUR%20HANDS%2012%20CASE%20SERIES.pdf.

[34] Yoo SJ, Thabit O, Lee W, Goo HW, Yim D, Ide H, van Arsdell GS (2015) Most peculiar hearts in your hands. Criss-cross, superoinferior, twisted, topsy-turvy, etc. What do they all mean? Web publication. https://doi.org/imib-chd.com/wp-content/uploads/morphology/2-mph-in-your-hands/MOST%20PECULIAR%20HEARTS%20IN%20YOUR%20HANDS%20Full%20Pages.pdf

[35] Messina M, Rigsby C, Deng J, Bi X, McNeal G (2013) 3D navigator-gated inversion recovery FLASH (Nav_IR_Flash) with blood pool contrast agent. Magnetom Flash 3/2013.

[36] Han F, Rapacchi S, Khan S, Ayad I, Salusky I, Gabriel S, Plotnik A, Finn JP, Hu P (2015). Four-dimensional, multiphase, steady-state imaging with contrast enhancement (MUSIC) in the heart: a feasibility study in children. Magn Reson Med.

[37] Samuel BP, Pinto C, Pietila T, Vettukattil JJ (2015). Ultrasound-derived three-dimensional printing in congenital heart disease. J Digit Imaging.

[38] Poterucha JT, Foley TA, Taggart NW (2014). Percutaneous pulmonary valve implantation in a native outflow tract: 3-dimensional DynaCT rotational angiographic reconstruction and 3-dimensional printed model. JACC Cardiovasc Interv.

[39] Yoo SJ, Seo JW, Lim TH, Park IS, Hong CY, Song MG, Kim SH, Choe KO, Cho BK, Lee HJ (1993). Hearts with twisted atrioventricular connections: findings at MR imaging. Radiology.

[40] Kurup HK, Samuel BP, Vettukattil JJ (2015). Hybrid 3D printing: a game-changer in personalized cardiac medicine?. Expert Rev Cardiovasc Ther.

[41] Kossivas F, Angeli S, Kafouris D, Patrickios CS, Tzagarakis V, Constantinides C (2012). MRI-based morphological modeling, synthesis and characterization of cardiac tissue-mimicking materials. Biomed Mater.

[42] Giannopouk AA, Chepelev L, Shikh A, Wang A, Dang W, Akyuz E, Hong C, Wake N, Pietila T, Dydynski PB, Mitsouras D, Rybicki FJ (2015) 3D printed ventricular septal defect patch: a primer for the 2015 Radiological Society of North America (RSNA) hands-on course in 3D printing, 3D Printing in Medicine 1:3 doi:10.1186/s41205-015-0002-4. 10.1186/s41205-015-0002-4 PMC6036609 30050972

[43] Gaetani R, Doevendans PA, Metz CH, Alblas J, Messina E, Giacomello A, Sluijter JP (2012). Cardiac tissue engineering using tissue printing technology and human cardiac progenitor cells. Biomaterials.

[44] Cheung DY, Duan B, Butcher JT (2015). Current progress in tissue engineering of heart valves: multiscale problems, multiscale solutions. Expert Opin Biol Ther.

[45] Mosadegh B, Xiong G, Dunham S (2015). Current progress in 3D printing for cardiovascular tissue engineering. Biomed Mater.

